# Continuous Infusion of Flumazenil in the Management of Benzodiazepines Detoxification

**DOI:** 10.3389/fpsyt.2021.646038

**Published:** 2021-03-18

**Authors:** Anna Benini, Rossella Gottardo, Cristiano Chiamulera, Anna Bertoldi, Lorenzo Zamboni, Fabio Lugoboni

**Affiliations:** ^1^Department of Diagnostics and Public Health, University of Verona, Verona, Italy; ^2^Forensic Toxicology Laboratory, Department of Diagnostics and Public Health, University of Verona, Verona, Italy; ^3^Department of Internal Medicine, Addiction Unit, Verona University Hospital, Verona, Italy

**Keywords:** benzodiazepine, flumazenil, withdrawal, high dose, detoxifcation

## Abstract

An effective approach in the treatment of benzodiazepine (BZD) overdosing and detoxification is flumazenil (FLU). Studies in chronic users who discontinued BZD in a clinical setting suggested that multiple slow bolus infusions of FLU reduce BZD withdrawal symptoms. The aim of this study was to confirm FLU efficacy for reducing BZD withdrawal syndrome by means of continuous elastomeric infusion, correlated to drugs plasma level and patients' compliance.

**Methods:** Seven-day FLU 1 mg/day subcutaneously injected through an elastomeric pump and BZDs lormetazepam, clonazepam, and lorazepam were assessed by HPLC-MS/MS in serum of patients before and after 4 and 7 days of FLU continuous infusion treatment. Changes in withdrawal severity were assessed by using the BZD Withdrawal Scale (BWS).

**Results:** Fourteen patients (mean age ± SD 42.5 ± 8.0 years, 5 male and 9 female), admitted to the hospital for high-dose BZD detoxification, were enrolled in the study. Serum FLU concentrations significantly decreased from 0.54 ± 0.33 ng/ml (mean ± SD) after 4 days of treatment to 0.1 ± 0.2 ng/ml at the end of infusion. Lormetazepam concentrations were 502.5 ± 610.0 ng/ml at hospital admission, 26.2 ± 26.8 ng/ml after 4 days, and 0 at the end of treatment. BWS values decreased during FLU treatment temporal period. FLU was well-tolerated by patients.

**Conclusions:** Elastomeric FLU infusion for BZD detoxification is a feasible administration device to maintain adequate, constant, and tolerated FLU concentrations for reducing BZD withdrawal symptoms.

## Introduction

Although benzodiazepines (BZDs) constitute one of the most broadly prescribed drug classes worldwide, the frequent and often inappropriate use is a problem that remains considerably underestimated by practitioners and most regulatory agencies (1). BZD can produce tolerance and dependence; thus, their use is recommended for a limited time (2). Surveys carried out in the 1990s in France, Germany, Italy, and the United Kingdom showed that 3.9% of hypnotic drug users and 3.2% of anxiolytic drug users had been taking a dose exceeding the recommended one (2–4). In Italy, about 7.5–10% of adult population are BDZ users, half of these being long-term users (LTU) with a diagnosis of BZD use disorder (5). Another study conducted in Italy showed that 14.0% of patients visiting general practitioners were taking BZDs, with 4.7% of the total sample being LTU, using BZDs daily for at least 12 months (6).

BZD tolerance was first reported in 1961 (7), but this phenomenon has been often obscured by the enthusiastic use of these drugs, which were able to replace barbiturates. The low toxicity coupled to a high potential of tolerance can lead to very high-dose misuse (8). From a clinical point of view, the only proposed solution of a gradual reduction of BZD is too simplistic. For long-term users, in general, if properly applied, gradually reducing the dosage works, but it is much less effective for high-dose users (2, 8, 9). This is worth mentioning because withdrawing from high doses of BZD carries significant risk for the health of the patient (2, 10).

It is in this area of HDUs that the use of flumazenil (FLU), used worldwide to treat the overdose of BZD, has been demonstrated as effective (9, 11–13). Experimental findings have shown that FLU acts as a BZD partial agonist with a weak intrinsic activity, when administered by slow intravenous infusion. While withdrawal symptoms may be brought on by the use of FLU, BZD-tolerant patients only reported mild symptoms (14, 15).

BZDs positively modulate γ-aminobutyric acid (GABA) through distinct binding sites on GABA_A_ receptors, and there is little variation among BZDs in pharmacodynamical factors such as selectivity and efficacy. Consequently, the choice of a particular BZD for clinical use is primarily based on pharmacokinetic features. Only one drug, flumazenil (FLU), is currently approved to reverse the effects of BZDs. FLU is a BZD partial agonist commonly used in the treatment of BZD overdose. Studies in chronic users who have discontinued BZDs suggested that multiple slow bolus infusions of FLU reduce the symptoms of BZD withdrawal when compared to placebo (9). The mechanism of FLU action remains, however, unclear: its action may facilitate the coupling of GABA_A_ and BZD receptor complexes, presumably by reversing the down-regulation/uncoupling that occurs with long-term BZD use (16). This mechanism is supposed to underlie FLU's weak agonist action and may explain its ability to attenuate BZD withdrawal symptoms (9). FLU does not antagonize the effects of other CNS sedative-hypnotics, such as ethanol, opioids, or general anesthetics (17).

FLU owns a rapid and extensive distribution phase with high volume of distribution and a second phase with fast metabolic elimination and short half-life (18). Its brief BZD-antagonism duration is due to a rapid hepatic elimination, determining its short half-life (60–90 min) and high plasma clearance (31–78 l/h). The low plasma protein binding of FLU (about 50%) does not limit its wide distribution (apparent distribution volume 0.6–1.6 l/kg) or its partly flow-dependent hepatic elimination (19, 20). Pharmacokinetic parameters of FLU do not change whether the drug is administered alone or in combination with other BZDs (18). For BZD detoxification, a viable method is the intravenous administration of FLU by using multiple bolus infusions either alone (14, 21) or in combination with tapering doses of BZDs (11).

The pharmacodynamical mechanisms of FLU are therefore crucial to determine its clinical effect, which could be achieved thanks to specific FLU infusion parameters in order to guarantee timing and extent of receptor occupancy (14). Thus, the choice of the most appropriate mode of delivery must be based on the correlation between FLU infusion parameters, plasma levels, and clinical endpoint. Our addiction unit has been employing FLU for high-dose BZD detoxifications since 2003, initially by means of endovenous continuous infusion administered by day. Such mode of delivery was both inconstant at maintaining adequate serum levels, being unfeasible for the night, and uncomfortable for the patient. In order to maintain constant serum concentration of FLU and to reduce modality of administration from multiple to single, we aimed to deliver FLU by slow subcutaneous infusion by using an elastomeric infusion pump at constant flow. In this study, we correlated the efficacy of continuous elastomeric FLU infusion on BZD withdrawal clinical endpoint to both drugs' (FLU and BZDs) plasma levels and, of equal importance, to patients' compliance and tolerance to treatment.

## Materials and Methods

This study was approved by the Ethical Review Board of the University Hospital (protocol number: 50771; prog. n. 683CESC). Informed consent was obtained from each subject.

### Subjects

Five male and nine female patients (mean age ± SD 42.5 ± 8.0 years), admitted to the hospital for BZD detoxification, were enrolled in the study (see Table 1 for patients' characteristics). The BZD use was stopped on day 1 of admission. The therapy with antidepressants, if any (Table 1), was maintained and continued after discharge.

**Table 1:** Patients' characteristics.

**Patient number**	**Gender (M/F)**	**Age (years)**	**Reported BDZ dosage at admission**	**Antide** **pressant**
1	M	38	LRZ 25 mg/day	Agomelatine
			CLO 2 mg/day	
2	M	44	LRM 75 mg/day	None
3	F	42	LRM 75 mg/day	Duloxetine
4	F	55	ALP 35 mg/day	Escitalopram
5	F	47	CLO 12 mg/day	Mirtazapine
6	F	52	LRM 25 mg/day	Venlafaxine plus agomelatine
			LRM 12 mg/day	
			DZP 100 mg/day	
			DZP 30 mg/day	
			FLZ 180 mg/day	
			TRZ 1.5 mg/day	
			LRZ 15 mg/day	
			ALP 4 mg/day	
			CLO 12 mg/day	
7	F	47	LRM 400 mg/day	Agomelatine
			DLZ 12 mg/day	
8	F	37	LRM 40 mg/day	Agomelatine
9	M	36	LRM 150 mg/day	None
10	F	43	LRZ 50 mg/day	Citalopram
11	F	31	LRM 100 mg/day	Paroxetine
12	F	30	LRM 75 mg/day	Sertraline
13	M	38	ALP 15 mg/day	Escitalopram
14	M	32	LRM 150 mg/day	Citalopram

*ALP, aprazolam; CLO, clonazepam; DLZ, delorazepam; DZP, diazepam; F, female; FLZ, flurazepam; LRM, lormetazepam; LRZ, lorazepam; M, male; TRZ, triazolam*.

All patients reported a history of BZD dependence according to the Diagnostic and Statistical Manual of Mental Disorders, Fifth Edition (DSM-5) criteria (22). Before hospitalization, all patients were interviewed by a physician to assess degree of BZD dependence and general health conditions. All patients had voluntarily contacted the Addiction Unit of Verona University Hospital and were aware of their BZD dependence.

Inclusion criteria were as follows: (i) age older than 18 years; (ii) diagnosis of BZD use disorder according to the DSM-5 criteria; (iii) BZD abuse lasting more than 6 months; and (iv) high dose of BZD abuse, meaning BZD intake exceeding at least five times the recommended daily amount (e.g., >50 mg in diazepam equivalents). Individuals were excluded if presenting the following: (i) current substance use disorder, defined as a history of illicit drug dependence or abuse within the previous 6 months; (ii) active medical illnesses or psychosis; and (iii) previous history of seizures, but not due to BZD withdrawal.

### Elastomeric Pump

Patients were treated with a solution containing 7 mg of flumazenil (Anexate®, Roche), available commercially in 0.5 mg/5 ml vials at pH = 4. The elastomeric pump (Infusor LV 1.5, code 2C1087K, Baxter S.p.A., Rome, Italy) was arranged with a maximum capacity of 250 ml and constant release of 1.5 ml/h for 7 days. The pump was connected to the patient's anterior abdominal wall via a butterfly needle inserted subcutaneously. The pump, releasing 1 mg of flumazenil every 24 h, was then placed in a small bag that could be carried attached to the belt or on the shoulder. Patients' tolerance for the infusion device was investigated on a daily basis, through clinical examination and interview.

Throughout the detoxification, FLU subcutaneous infusion (FLU-SI) was associated with therapeutic doses of clonazepam, orally administered every day in the evening and gradually tapered from 6 mg on the 1st day to 0.5–2.0 mg on the last day of treatment. The different speed in the tapering of clonazepam was due to clinical criteria, in particular we considered the quality of sleep and the intensity of withdrawal symptoms. In this way, at the end of hospitalization, 3/14 patients were discharged with no clonazepam, and 11/14 (78.6%) patients were discharged with a low dose of clonazepam ranging from 0.5 to 2.0 mg/day; these patients were recommended to gradually taper it in a few weeks (8). Unfortunately, patients were not followed-up as outpatients, and we cannot be sure whether they succeeded in tapering and eventually stopping clonazepam.

Ten days prior to the admission, anti-epileptic prophylaxis (1 g/day valproic acid or levetiracetam) was given to all patients in order to prevent seizures during treatment. Anti-epileptic treatment was maintained during the hospital stay and for further 20–40 days after discharge.

Patients under concurrent treatment with antidepressant (12/14 patients, see Table 1) were maintained under this pharmacotherapy.

### Sampling Protocol

Blood samples were collected without anticoagulant at the moment of admission, after 4 days of FLU treatment, and at the end of the 7 days of treatment, before discharge from the addiction unit.

Samples were centrifuged (3,000 rpm, 10 min) and sera were frozen at −80°C until HPLC-MS analysis.

### Flumazenil and Main BZD Concentration Analysis

Blank serum samples, used for the development and validation of the procedure, were obtained from healthy volunteers abstinent from any drug during the week before sampling. A 250-μl aliquot of serum was added to an equal volume of 0.1 M phosphate solution (pH 8.8), and the mixture was spiked with the IS (diazepam-D5) to have a final concentration of 40 ng/ml. The mixtures were added with 1.5 ml of ethyl acetate, then extracted by vortex-mixing for 1 min, and centrifuged at 4,000 rpm for 15 min. The organic phase was then evaporated to dryness under nitrogen stream and the residue dissolved in 50 μl of ultrapure water.

The determination of FLU and lormetazepam was obtained by using a model 1290 UHPLC coupled to a model 6450 triple quadruple mass spectrometer (Agilent Technologies, Waldbronn, Germany) operating in positive ionization mode. Gradient elution was performed on a UHPLC ZORBAX Eclipse reversed-phase column (RRHD 2.1 mm × 100 mm, 1.8 μm) (Agilent) by mixing 5 mM aqueous ammonium formate containing 0.01% formic acid (eluent A) and acetonitrile added with 0.01% formic acid at a flow rate of 0.5 ml/min (eluent B) from 10 to 95% B in 7 min. The analyses were performed in multiple reaction ion monitoring (MRM) mode using the following ion transitions: FLU 304 217, 232, and 258 (collision energy: 20 eV); lormetazepam 335 317, 289, and 177.0 (collision energy: 20 eV); and diazepam-D5 290 262 (collision energy: 27 eV).

Method was linear in the concentration range of 78–5,000 pg/ml for FLU and of 3–200 ng/ml for lormetazepam. Lower limit of quantification (LLOQ) corresponded to 78 pg/ml for FLU and 3 ng/ml for lormetazepam.

Precision (% CV) of the assay was ≤9.8% for both the analytes, whereas the inter-assay accuracy was ≤3.8 and ≤4.7%, respectively. The accuracy and CVs for day-to-day tests resulted always below 7.93%.

### Withdrawal Assessment

A Benzodiazepine Withdrawal Scale (BWS) form exploring withdrawal symptoms (33 items each with a score of 0–4 from best to worst) was given to each patient for daily report (23).

### Statistical Analysis

Statistical analysis was performed using the software Graph Pad PRISM version 6.0. The results were expressed as mean ± standard error of the mean (SEM). Student's *t*-test was utilized for statistical analysis by comparing different treatment times of the same group of patients.

## Results

**Figure 1:**
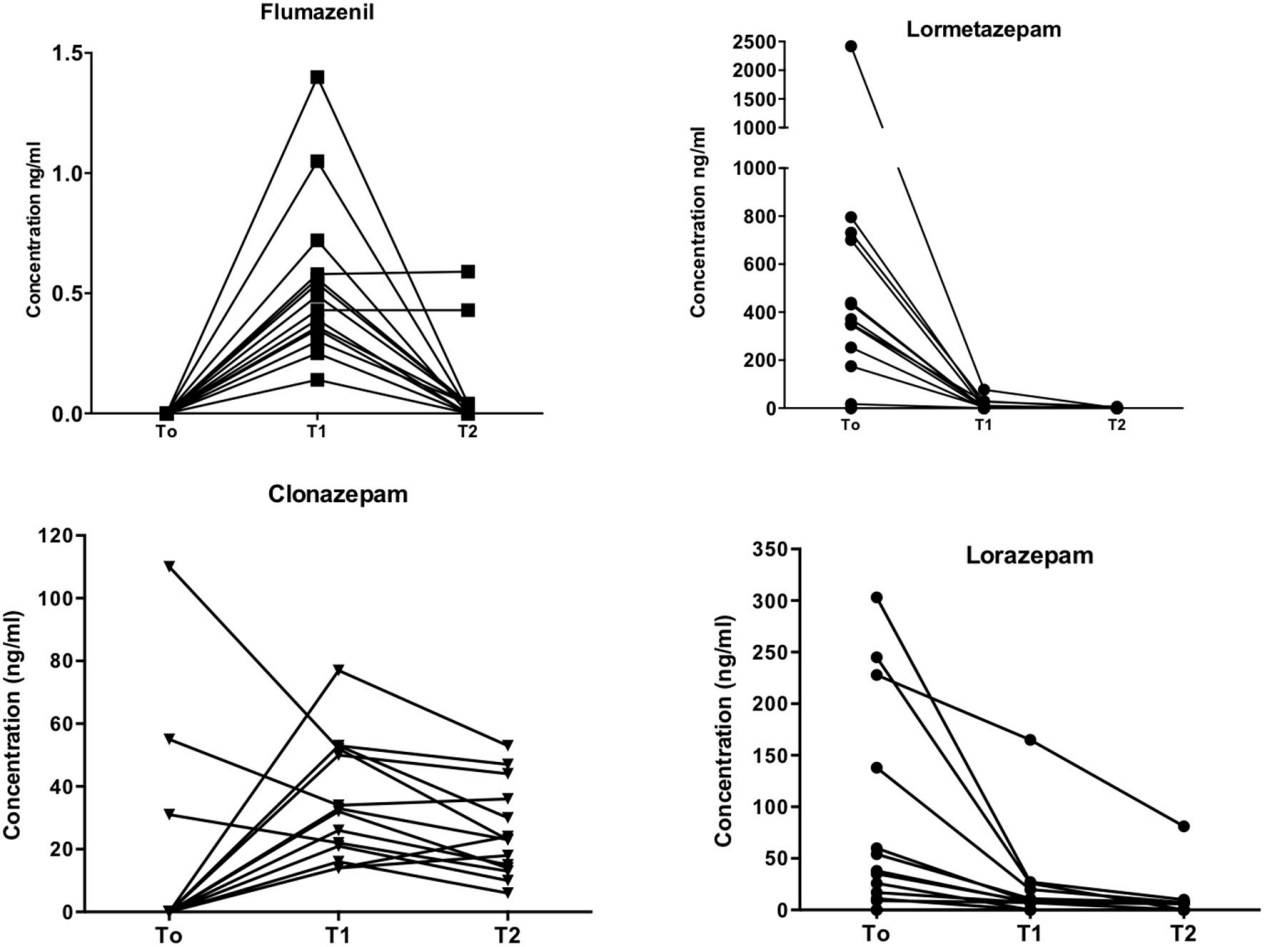
Drugs plasma levels. Individual plasma levels (ng/ml; ordinates) for flumazenil, lormetazepam, clonazepam, and lorazepam at different time-points (abscissa), i.e., at admission (T0), four (T1) and seven days after flumazenil start of elastomeric infusion.

Drug plasma levels are shown in Figure 1. Plasma FLU concentrations were 0.54 ± 0.089 ng/ml (mean ± SEM) at T1 after 4 days of continuous subcutaneous infusion, ranging from 0.14 to 1.4 ng/ml. Values recorded at T2 (end of therapy) were 0.09 ± 0.05 ng/ml, with FLU concentrations below limits of detection in 10 patients out of 14.

Lormetazepam (LRM) levels were 502.5 ± 163.0 ng/ml at T0 baseline. A significant decrease (11.2 ± 5.7 ng/ml; *p* = 0.008) in LRM levels was recorded at T1 and 0.43 ± 0.43 ng/ml at T2. High LRM plasma levels recorded at T0 are in agreement with patients' self-report of BZD use at admission, whereas low T1 and T2 levels confirmed compliance to detoxification treatment.

Lorazepam (LRZ) levels showed a similar pattern, with high initial plasma concentrations (83.1 ± 27.4 ng/ml), then a significant decrease to 20.4 ± 11.4 ng/ml (*p* = 0.01) at T1 and 9.4 ± 5.6 ng/ml at T2 after 7 days of FLU administration.

Clonazepam (CLN) plasma levels were low at T0 (14.0 ± 8.6 ng/ml), 35.5 ± 5.0 ng/ml at T1, and 25.4 ± 3.9 ng/ml at T2. Note that three patients were treated with CLN before hospital admission (see Table 1).

**Figure 2:**
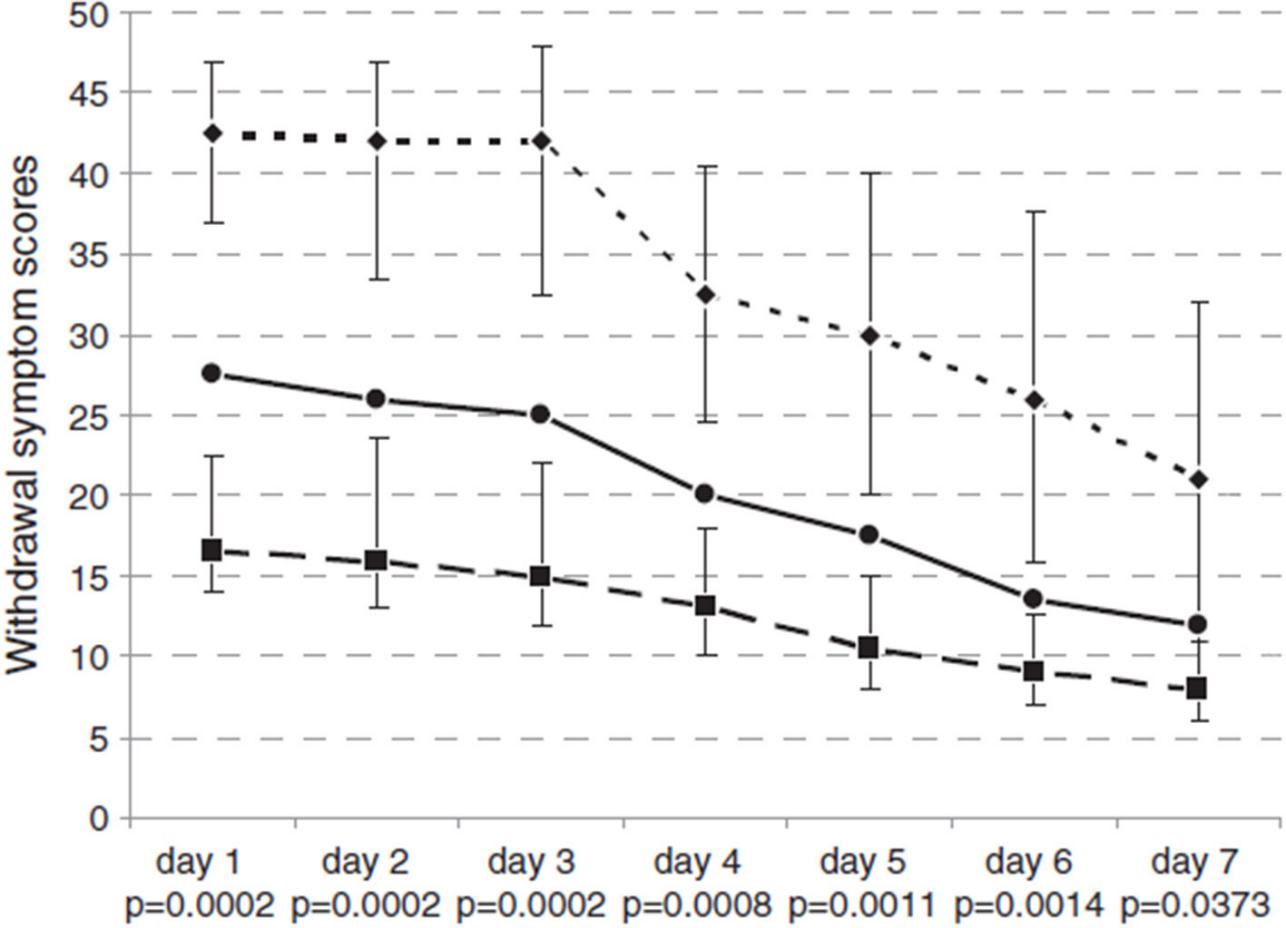
Withdrawal Symptom Scores from day 1 to day 7 of FLU-SI treatment.

According to different BZD behaviors, BWS showed a decrease from 26.4 to 17.7 points, as portrayed in Figure 2. During the treatment, 10/14 subjects (71.4%) completed the Benzodiazepine Withdrawal Scale (BWS) with scores ranging from 0 to 132 on a daily basis, in order to subjectively assess their withdrawal symptoms. Four out of 14 patients could not complete the BWS. As shown in Figure 2, BWS improved significantly during FLU treatment in all subjects. No major event (i.e., convulsive crisis) occurred.

The elastomeric pump was well tolerated by patients. Since FLU is further diluted in a saline solution inside the device, no skin irritation around the insertion of the needle was noticed. Since elastomeric pumps are light and compact, patients appreciated the freedom of movement and rated them as painless, safe, and comfortable, with no bound to the pump and respecting the privacy about the therapy, whereas nurses acknowledged they required less time to manage them.

## Discussion

BZD represents a class of drugs characterized by low acute toxicity even at high doses in the absence of any concurrent drug abuse such as alcohol and opioids (2). Literature data on the toxicity of high-dose BZD are old and mostly based on anecdotal case reports. The lack of clinical studies and the high tolerability of these drugs have produced the erroneous perception that the administration of high doses of BZD for a prolonged time, although not recommended, could be not harmful. However, several complications have been associated to chronic BZD consumption, such as memory and attention deficit, inability to learn, increased risk of falls, road accidents, depression, and reduced quality of life (Lugoboni DAD 2014). Thus, although the prolonged use of high dose of BZD seems not to induce liver toxicity, it remains a serious health concern (24). The severe discomfort experienced by patients stopping long-term BZD use led to the development of treatment strategies for discontinuing these medications (1, 10). The common management of BZD withdrawal syndrome includes, either individually or in combination: (i) a gradual tapering of the drug; (ii) switching to an equivalent dose of a long half-life BZD before tapering withdrawal (10, 25); and (iii) adding medications prior to detoxification and continuing those medications after BZD discontinuation (1, 10). A potential approach is the abrupt discontinuation of the medication and a rapid BZD detoxification using FLU. FLU is commonly used in the treatment of BZD overdose; it is usually considered a BZD antagonist (9). When compared to placebo, bolus infusion of flumazenil (1 mg in 5 min) produced effects similar to BZD withdrawal in BZD users (23, 26). Nonetheless, results of studies in chronic BZD users who have discontinued BZD use suggest that multiple slow bolus infusions of flumazenil reduce the symptoms of withdrawal (9, 11, 21, 27).

Subcutaneous route of FLU administration was previously described only in three patients (14), suggesting the usefulness of this route for its excellent tolerability, efficacy, and improvement on measure of psychological distress. According to these data, we decided to administer FLU by subcutaneous route utilizing elastomeric pumps normally used for pain control in cancer patients or, more recently, for continuous infusion of antibiotics (28) or for treatment of idiopathic hypersomnia (29).

To our knowledge, the results present in this paper are the first data of FLU serum concentrations following subcutaneous infusion by elastomeric pump described in literature. FLU serum concentrations were low, but consistent with data of FLU administered by i.v. route (14).

FLU is characterized by short half-life (0.8–1.2 h) (30) and requires repeated doses or continuous infusion to reverse BZD overdose. In spite of its low lipophilicity, FLU has a large volume of distribution, and its weak binding to plasma proteins explains its rapid distribution. Moreover, FLU is extensively metabolized by hepatic cytochromes P450 3A4, 3A5, and 2C9 and readily eliminated. Maximum brain concentrations are reached 5 to 8 min after i.v. administration (31).

Subcutaneous administration of flumazenil eliminates some problems with first-pass hepatic metabolism observed orally and is likely to facilitate better absorption. Subcutaneous administration also provides continuous dosing, which would be hard to achieve with oral or sublingual administration, and the slow absorption may abrogate side effects related to high serum concentrations. The subcutaneous route is easier to establish than the intravenous administration, and there is no risk for patient's veins. Study data suggested that flumazenil administered by the s.c. route might have equitable clinical benefits to i.v. administration, but it might be superior in that it requires less clinical monitoring and is likely associated with less equipment problems (i.e., dislodged or blocked i.v. needle/line) and adverse events (i.e., venous tissue irritation). These advantages, as well as an improved patient mobility over the treatment period, will also likely result in increased patient satisfaction (9, 14).

The subcutaneous route of administration may be associated to the absence of adverse events associated with i.v. FLU administration. In fact, our patients did not report any kind of adverse events such as those frequently reported during or after FLU administration (8, 14, 32).

Our results demonstrated low and constant serum concentrations during all treatment and a prompt decrease nearly to 0 at the end of treatment, protecting patients from peak serum levels. We utilized an elastomeric infusion pump mostly utilized in our hospital for analgesic purposes.

Several elastomeric pumps are commercially available, and they are calibrated in different conditions, including operating temperature and pressure, viscosity of fluid, backpressure, and time recommended between filling of the device and beginning of the infusion. All of these factors affect the infusion rate of pumps. Elastomeric infusion pumps are feasible to use and less bed bounding for patients, although a little less precise than other pumps.

Moreover, Höjer et al. (33) studied the stability of infusion solutions of flumazenil in concentrations of 1.0 and 5.0 μg/ml stored for periods of up to 9 months and concluded that the stability of flumazenil in infusion solution was satisfactory.

Importantly, serum levels of other BZDs (such as LRM and LRZ) are 0 after 4 days of FLU administration, proving both the efficacy of FLU and patients' compliance despite the elevated BZD plasma levels measured at the beginning of the treatment. The good patients' compliance was confirmed by CLN concentrations in serum that showed a trend to decrease after 7 days. Most interestingly, during the detoxification process, all patients reported low levels of craving for BZD, which might represent a rarely seen feature in the spectrum of drug detoxification. According to previous studies, high-dose BZD chronic use determines a severe impairment of psychological, physical, and social functioning, along with a significant reduction of quality of life (34, 35).

The main limitation to this study is the lack of a follow-up phase to determine whether all patients were successfully able to taper and suspend clonazepam and to assess the relapse rate. Another limitation of the study is its monocentric design. The problem is not new. Although more than 30 years have passed since the first studies of the efficacy of FLU in the treatment of addiction to high doses of BZD, to our knowledge, there are no more than five centers worldwide offering this treatment. This continues to represent a major obstacle to the definition of more shared and standardized protocols. Currently, FLU protocol is the same for all patients, regardless of sex, age, BMI, and BZD daily intake. Future prospects should include further investigations of the individual variables and clinical outcomes in order to individualize the detoxification therapy.

## Data Availability Statement

The raw data supporting the conclusions of this article will be made available by the authors, without undue reservation.

## Ethics Statement

The studies involving human participants were reviewed and approved by CE 292CESC. The patients/participants provided their written informed consent to participate in this study.

## Author Contributions

ABen and RG equally contributed for the analytes'plasma measurements. ABen, CC, and ABer contributed to the manuscript's draft and proofreading. LZ processed the statistical analysis and FL followed the clinical part and designed the study. All authors contributed to the article and approved the submitted version.

## Conflict of Interest

The authors declare that the research was conducted in the absence of any commercial or financial relationships that could be construed as a potential conflict of interest.
